# A Novel Modified Two-Lobe Holmium Prostate Enucleation Technique: Demirtaş–Erciyes Enucleation Prostatectomy

**DOI:** 10.7759/cureus.22144

**Published:** 2022-02-11

**Authors:** Abdullah Demirtaş, Şevket T Tombul, Gökhan Sönmez, Türev Demirtaş

**Affiliations:** 1 Urology, Erciyes University, Kayseri, TUR; 2 History of Medicine and Ethics, Erciyes University, Kayseri, TUR

**Keywords:** prostate, laser enucleation, prostatectomy, anatomic endoscopic prostate enucleation, holep

## Abstract

Objective

Endoscopic enucleation of the prostate has evolved and became popular for the surgical treatment of benign prostatic hyperplasia (BPH) during the last decade. Different surgical techniques have been described so far. We hereby described a new modified two-lobe technique for urologists who are inexperienced in endoscopic enucleation. We aimed here to present the data on a learning curve of this stepwise technique named Demirtaş-Erciyes Enucleation Prostatectomy (DEEP): reverse S-J incision technique and its postoperative outcomes.

Material and methods

The study included 102 patients who underwent holmium laser enucleation of the prostate (HoLEP) with the DEEP technique between October 2020 and December 2021. Demographic, preoperative, and postoperative variables were recorded. The operation was performed with a 150 W holmium laser system (Quanta System, Varese, Italy) with cutting and coagulation settings of 2J × 50 Hz with virtual basket mode and 2J × 12 Hz in bubble blast mode, respectively. Bladder irrigation was done for one day, and then, on the next day, the urethral catheter was removed. Postoperatively, uroflowmetry studies, continence status, and ejaculation status were recorded during follow-up. The data of all patients were divided into two groups (first 51 and final 51 patients). All variables were analyzed between two groups.

Results

The mean age of the patients was 68.48±8.74 years. The median Charlson Comorbidity Index (CCI) score was 3. The median International Prostate Symptom Score (IPSS) and International Index of Erectile Function (IIEF) values ​​were 26 (10-35) and 10 (0-25), respectively. Of the patients, 60.8% had Foley catheters due to urinary retention. The median anesthesia time, laser time, enucleation time, morcellation time, and enucleated tissue amount were 102.5 minutes, 17 minutes, 25 minutes, 20 minutes, and 50 g, respectively. Enucleation was performed in two stages in five patients due to bigger prostate volume or incomplete morcellation. The median catheter removal time was 48 hours. In six patients, the postoperative catheterization time was prolonged due to hematuria. The median increase in Qmax was 19.35 mL/second. The overall complication rate was 5.9%, which were all Clavien grade II. Enucleation time, laser time, and anesthesia time were significantly lower in the last 51 patients.

Conclusion

DEEP enucleation technique seems to provide effective and safe postoperative results for beginners in prostate enucleation.

## Introduction

Benign prostatic hyperplasia (BPH) is the most common disease-causing lower urinary tract symptoms in aging men [1,2]. Although medical treatment is usually sufficient in mild BPH cases, surgical intervention may be necessary in refractory cases. Monopolar or bipolar transurethral resection of the prostate (TURP) is recommended in prostates weighing 30-80 g, whereas open prostatectomy (OP) is essential in prostates weighing over 80 g. Nevertheless, endoscopic enucleation, which was described long ago, has become a widely popular technique, particularly in the form of holmium laser enucleation of the prostate (HoLEP), due to the developments in both laser and endoscopic systems [3-5]. HoLEP has been demonstrated to provide more effective perioperative and postoperative results compared to both TURP and OP in the short and medium terms. Studies have also shown that HoLEP is highly advantageous since it leads to fewer intraoperative complications, its catheter can be withdrawn in the early period, it requires relatively less blood transfusion in the postoperative period, and most importantly, it provides a high level of patient satisfaction [6,7].

To date, various HoLEP techniques have been developed, including en bloc, two-lobe, three-lobe, and bladder neck-preserving techniques. In two-lobe techniques, various incisions are made to separate the mucosa from the sphincter. Nevertheless, to our knowledge, no stepwise technique that could be utilized by urologists unexperienced in endoscopic enucleation has been described in the literature [8-10]. The modified two-lobe technique for the surgeons with no experience of enucleation and in which the enucleation starts from the median lobe and left lobe and then right lobe was defined here. This study aimed to present data on the learning curve obtained by the stepwise application of Demirtaş-Erciyes Enucleation Prostatectomy (DEEP): reverse S-J incision technique, which is a modified two-lobe enucleation technique, and also to present the postoperative outcomes of this technique.

## Materials and methods

The study reviewed the prospective data of 102 patients who underwent HoLEP with the DEEP technique between October 2020 and December 2021 in Erciyes University Medical School Urology Clinic. Demographic and clinical data including age, gender, body mass index (BMI), preoperative prostate-specific antigen (PSA) values, routine biochemical and blood tests, total prostate volume (TPV) and transition zone volume (TZV) determined by transrectal ultrasound (TRUS), uroflowmetry results, preoperative International Prostate Symptom Score (IPSS) and International Index of Erectile Function (IIEF) scores, previous BPH treatments, and the requirement of ureteral catheter insertion were recorded for each patient. In all patients, TRUS was performed preoperatively. All patients were operated on after obtaining clean urine culture. All surgical procedures were performed under general anesthesia. Intraoperative prophylactic antibiotics; the duration of anesthesia, enucleation, and laser application; the amount of laser energy utilized; the amount of saline irrigation used during the procedure; the duration of morcellation; the amount of tissue removed; and intraoperative and postoperative complications were recorded. Complications were evaluated according to the Clavien-Dindo classification [11]. In the postoperative period, irrigation termination time, catheter removal time, length of hospital stays, postoperative biochemical and blood counts, and temporary or permanent urinary incontinence state in the first postoperative month were verbally questioned, and the uroflowmetry values ​​were recorded. The preoperative, perioperative, and postoperative variables of the first 51 (group I) and the last 51 (group II) patients were compared and analyzed to detect if the experience of the surgeon affect them. Ongoing antiaggregant and anticoagulant therapies were discontinued during the preoperative period in line with consultation with relevant departments, and low-molecular-weight heparin was initiated as needed. Informed consent was obtained from each patient. All operations were performed by the same surgeon under general anesthesia, and he is inexperienced with holmium laser enucleation at the beginning.

Surgical procedure and description of the technique

The HoLEP procedure was performed using the two-lobe DEEP: inverted S-J incision technique. A 26 Fr resectoscope (Karl Storz Endoscopy, Tuttlingen, Germany) and a 150 W holmium laser system (Quanta System, ​​Varese, Italy) were used with cutting and coagulation settings of 2J × 50 Hz with virtual basket mode and 2J × 12 Hz in bubble blast mode, respectively. The DEEP technique consists of 12 steps, which are performed following routine cystourethroscopy (Video 1):

Step 1: A reverse S (Ƨ) incision is made circumferentially from the 11 o'clock position to the 5 o'clock position near the verumontanum, and the incision is lengthened down to the level of the capsule.

Step 2: An incision is made between the 7 o'clock position near the bladder neck and the upper end of the Ƨ incision created in the previous step, and the incision is lengthened down to the level of the capsule. The right and median lobes are separated from each other.

Step 3: The apical part of the left lobe is incised from the 5 o'clock position near the verumontanum inferiorly to the anterior commissure and then separated from the sphincter.

Step 4: At 12 o'clock, the anterior commissure is incised up to the bladder neck. The right and left lobes are separated from each other.

Step 5: The left lobe is enucleated from the verumontanum to the bladder neck in a top to bottom fashion. The median lobe is enucleated from the verumontanum to the bladder neck in a right to left fashion.

Step 6: The left lobe together with the median lobes are separated from the bladder neck.

Step 7: The apex of the right lobe is incised with a J incision from the 11 o'clock position of the Ƨ incision to the 7 o'clock around the verumontanum. The incision is lengthened down to the level of the capsule.

Step 8: The right lobe is incised up to the anterior commissure. This incision is deepened to the capsule, and the right separated from the sphincter.

Step 9: The right lobe is enucleated from the verumontanum to the bladder neck in a top to bottom fashion.

Step 10: The right lobe is separated from the bladder neck.

Step 11: Morcellation.

Step 12: In cases that had a total urethral instrumentation period of over 90 minutes in the first six steps, a second session that consisted of step 7 and later steps was performed.

After enucleation, tissues were removed using a mechanical morcellator (Karl Storz Endoscopy, Tuttlingen, Germany). In this technique, there are no steps regarding the protection of the bladder neck.

**Video 1:** A novel modified two-lobe holmium prostate enucleation technique: DEEP DEEP: Demirtaş-Erciyes Enucleation Prostatectomy

Statistical analysis

Data were analyzed using Statistical Package for the Social Sciences (SPSS) version 22.0 (IBM Corporation, Armonk, NY, USA). The distribution patterns of data were determined using the Shapiro-Wilk test and histogram plots. Continuous variables with normal distribution were expressed as mean±standard deviation (SD), and variables without normal distribution were expressed as median (minimum-maximum). Categorical variables were expressed as percentages (%). Comparison of variables in two groups was made using the Mann-Whitney U test or Chi-square test as appropriate.

Ethical approval and funding

The study was approved by Erciyes University Clinical Research Ethics Committee (approval number: 2019/666) and was financially supported by Erciyes University Scientific Research Projects Coordination Unit (project number: TSG-2020-9673).

## Results

The mean age of the patients was 68.48±8.74 years. The median Charlson Comorbidity Index (CCI) score was 3 (1-7). The median IPSS and IIEF values ​​were 26 (10-35) and 10 (0-25), respectively. A urethral catheter had been inserted preoperatively in 60.8% (62/102) of the patients. Preoperative antibiotic treatment was initiated in 24 (25.5%) patients due to bacterial growth in urine culture. Abnormal rectal findings were detected in 15 (14.7%) patients. Prostate adenocarcinoma (Gleason score 3+3 and 4+5) was detected in only two patients in preoperative prostate biopsy. Both patients preferred radiotherapy as the definitive treatment. Before radiotherapy, patients underwent endoscopic prostate enucleation due to lower urinary tract symptoms. Other demographic and preoperative and postoperative clinical characteristics of the patients are summarized in Table 1.

**Table 1:** Demographic and clinical characteristics BMI: body mass index; PSA: prostate-specific antigen; TPV: total prostate volume; TZV: transition zone volume; UTI: urinary tract infection; IPSS: International Prostate Symptom Score; IIEF: International Index of Erectile Function

	Group I (first 51 patients)	Group II (final 51 patients)	Total (N = 84)	p
Age (years)	67.8±8.37	69.16±9.13	68.48±8.74	0.437
BMI (kg/m^2^)	27.82±3.29	27.14±3.36	27.40±3.30	0.302
PSA (ng/mL)	7.88±7.33	9.27±8.93	8.57±8.15	0.396
TPV (mm^3^)	76.40 (25-375)	78.18 (30-200)	76.40 (25-375)	0.799
TZV (mm^3^)	45.40 (20-217.3)	49.60 (20-147.3)	47 (20-217)	0.527
Preoperative catheterization	32/51 (62.7%)	30/51 (58.8%)	62/102 (60.8%)	0.685
Preoperative UTI	8/51 (15.7%)	16/51 (31.4%)	24/102 (23.5%)	0.062
IPSS	30 (10-35)	26 (10-35)	26 (10-35)	0.157
IIEF	10 (0-23)	10 (0-25)	10 (0-25)	0.449
Anesthesia time (minute)	120 (45-240)	90 (30-270)	102.5 (30-270)	0.004
Laser time (minute)	21 (8-75)	15 (3-42)	17(3-75)	<0.0001
Enucleation time (minute)	40 (7-105)	18 (8-60)	25(7-105)	<0.0001
Morcellation time (minute)	25 (10-100)	18(10-100)	20 (10-100)	0.178
Tissue removed (g)	58 (5-220)	45 (5-266)	50 (5-266)	0.085
Irrigation fluid (L)	30 (9-102)	33 (8-90)	33 (8-102)	0.976
Postoperative irrigation time (hour)	24 (12-360)	24 (20-48)	24 (12-360)	0.965
Catheterization time (hour)	36 (16-360)	48 (24-144)	48 (16-360)	0.005
Hospitalization (hour)	48 (24-384)	48 (24-144)	48 (24-284)	0.111
Hemoglobin change	-0.80 (-4.74 – 1.90)	-0.50 (-0.4 – 2.60)	-0.65 (-4.5 - 2.60)	0.177
Qmax elevation	20.9 (8.5-40.2)	18.70 (4.3-26.7)	19.35 (4.3-40.2)	0.002
Postoperative stress incontinence	17/51 (33.3%)	17/51 (33.3%)	34/102 (33.3%)	1
Retrograde ejaculation	100%	100%	100%	N/A

In five (4.9%) patients, the DEEP procedure was performed in two sessions. In three of these patients, right lobe enucleation was performed in the second session due to large prostate volume and bleeding. In the remaining two patients, the enucleation procedure was completed in the first step, while morcellation could not be completed in the first session since some of the enucleated tissues were fibrotic and the surgical procedure time was prolonged. In these patients, morcellation was completed by performing a second cystoscopy two days later. DEEP was performed in the first session in the remaining 97 (95.1%) patients. Urethral catheterization time was prolonged in six (5.9%) patients due to postoperative bleeding. One of these patients had platelet dysfunction due to hematological malignancy, while in the other five patients, antiaggregant and/or anticoagulant therapy, which had been discontinued due to comorbidities, had to be restarted in the early period, and prolonged hematuria occurred secondary to these treatments. The ongoing antiaggregant/anticoagulant treatments were regulated, and hematuria was regressed with bladder irrigation. No additional endoscopic intervention was required for these patients due to the presence of hematuria. However, the patients required blood transfusion due to a decrease in hemoglobin level. No complication was observed in any patient, except for the Clavien grade II complication observed in six (5.9%) patients.

A significant relationship was found between the two groups regarding the decrease in the duration of anesthesia, laser use, enucleation, and the increase in the experience of the surgeon, whereas no significant difference was found concerning other preoperative, perioperative, and postoperative factors. Also, as seen in Table 1, longer catheterization time and less increase in Qmax were detected in the last 51 patients; this is because more comorbid patients were present in the last 51 patients. On the other hand, in line with the increase in surgical experience, the enucleation time approached the laser usage time.

The median increase in the Qmax value in the postoperative controls was 19.35 (4.3-40.2) mL/hour. Stress incontinence was observed in 34 (33.3%) patients following the removal of the catheter in the early postoperative period. These patients were recommended to perform pelvic floor exercises, and none of them had ongoing stress incontinence at the first month postoperative follow-up. Permanent incontinence did not develop in any of the patients postoperatively, whereas all patients had retrograde ejaculation in the postoperative period. No urethral stricture developed in patients who completed the 12-month follow-up.

## Discussion

Since its introduction, HoLEP has become increasingly popular in clinical practice. Additionally, different techniques with different learning curves have been identified. On the other hand, enucleation techniques such as HoLEP have begun to change the gold standard in the surgical treatment of BPH [3,4,12,13]. In the present study, HoLEP was performed with the DEEP technique, and the median duration of anesthesia, enucleation, and laser usage was 102.5, 25, and 17 minutes, respectively. Additionally, the median amount of tissue removed was 50 g, and the urethral catheter was removed at a median period of 48 hours following one-day postoperative bladder irrigation. Although the decrease in postoperative hemoglobin level was statistically insignificant (median: 0.65 g/dL), blood transfusion was required in only six patients. In the postoperative period, the rate of stress incontinence was 33.3%, which remarkably regressed with Kegel exercises at the end of the first month. The median increase in Qmax value, which is an objective marker of the relief of voiding symptoms, was 19.35 mL/second. The increase in Qmax was about 334%. However, retrograde ejaculation was observed in all patients after the procedure.

In a recent randomized controlled study that compared HoLEP and plasmakinetic enucleation of the prostate (PKEP), the mean surgical time was 66.56 minutes, the mean enucleation time was 53.68 minutes, the mean amount of tissue removed was 53.93 g, and the mean morcellation time was 12.89 minutes in the laser arm. On the other hand, the mean bladder irrigation time and mean time to catheter removal were reported as 19.56 and 78.45 hours, respectively, and the rate of retrograde ejaculation was reported as 37.10%. However, the enucleation technique used in the study was not specified [14]. Another recent study compared different enucleation techniques and reported that the enucleation time, morcellation time, and amount of tissue removed in the two-lobe technique were 50 minutes, 10 minutes, and 40 g, respectively [15]. A meta-analysis conducted by Hartung et al. compared thulium laser enucleation of the prostate (ThuLEP) and HoLEP and reported that, in the HoLEP technique, the catheter was removed within a mean period of two days, and the patients were discharged in 2-2.5 days [16]. In our study, the mean morcellation time was significantly higher when compared to those reported in the literature. This difference could be attributed to the ineffective use of morcellation equipment. In contrast, the other perioperative data in our study seem to be compatible with those reported in the literature.

He et al. reported that transient urinary incontinence was observed in 7.9% of the patients and none of the patients required blood transfusion after the administration of HoLEP [17]. In another similar randomized controlled study, Xu et al. reported that, with the two-lobe HoLEP method, no postoperative transfusion was performed in any patient, transient urinary incontinence was observed in 1.07% of the patients, and the retrograde ejaculation rate at six months was 33.3% [18]. Placer et al. reported the retrograde ejaculation rate to be 70.3% in their HoLEP series [19]. In our study, the transfusion, stress incontinence, and retrograde ejaculation rates were significantly higher than those reported in the literature [18-20]. Among the studies in the literature, those that reported no blood transfusion did not clearly state whether the patients received anticoagulant/antiaggregant therapies [18-20].

In our series, transfused patients had thrombocyte dysfunction or other comorbidities that required early anticoagulant initiation. The definition of incontinence has not been given clearly in the literature. In the present study, stress incontinence, which was found to have a prevalence of 33.3% in our series, was defined as uncontrolled urinary incontinence due to increased intraabdominal pressure following catheter removal, and all patients recovered completely after one month of pelvic floor exercises. Unlike the literature data, the retrograde ejaculation rate in our study was 100%. On the other hand, no specific ejaculation-preserving technique was used in the described technique; therefore, retrograde ejaculation was inevitable in all patients.

The primary limitations of the study were its single-arm design and the relatively small number of patients. However, the HoLEP method, when used with the DEEP technique, seems to be sufficiently reliable and safe for urologists who are new to enucleation surgery. Further large-scale double-armed randomized studies to be conducted with other enucleation techniques are needed to substantiate our findings.

In our study, no significant difference was found between the two groups concerning functional outcomes and complications. However, a significant improvement was observed in group II concerning perioperative variables (durations of anesthesia, laser usage, and enucleation). These findings implicate that, after using this technique in more than 50 cases, perioperative variables reached a plateau, i.e., the learning curve was completed. In the meta-analyses reported in the literature, it has been shown that the learning curve for HoLEP surgery is completed in 30-50 cases [12,20,21].

## Conclusions

The modified two-lobe technique defined here provides effective perioperative and postoperative results. It also enables surgeons to complete enucleation in two steps whenever necessary. Like other techniques previously defined, it has similar learning curves. Although the need for comparative studies on two-lobe techniques is obvious, the DEEP enucleation technique seems to provide effective and safe postoperative results for beginners in prostate enucleation.
